# The Roles of Syncytin-Like Proteins in Ruminant Placentation

**DOI:** 10.3390/v7062753

**Published:** 2015-06-05

**Authors:** Yuki Nakaya, Takayuki Miyazawa

**Affiliations:** 1 Department of Infectious Diseases, Kyoto Prefectural University of Medicine, 465 Kawaramachi-hirokoji-Kajiicho, Kamigyo-ku, Kyoto 602-8566, Japan; 2 Laboratory of Signal Transduction, Department of Cell Biology, Institute for Virus Research, Kyoto University, 53 Shogoin-Kawaharacho, Sakyo-ku, Kyoto 606-8507, Japan

**Keywords:** ruminants, endogenous retrovirus, envelope glycoprotein, fematrin-1, syncytin-rum1, placenta, cell-to-cell fusion

## Abstract

Recent developments in genome sequencing techniques have led to the identification of huge numbers of endogenous retroviruses (ERV) in various mammals. ERVs, which occupy 8%–13% of mammalian genomes, are believed to affect mammalian evolution and biological diversity. Although the functional significance of most ERVs remains to be elucidated, several ERVs are thought to have pivotal roles in host physiology. We and other groups recently identified ERV envelope proteins (e.g., Fematrin-1, Syncytin-Rum1, endogenous Jaagsiekte sheep retrovirus Env) that may determine the morphogenesis of the unique fused trophoblast cells, termed trinucleate cells and syncytial plaques, found in ruminant placentas; however, there are still a number of outstanding issues with regard to the role of ERVs that remain to be resolved. Here, we review what is known about how these ERVs have contributed to the development of ruminant-specific trophoblast cells.

## 1. Introduction

Endogenous retroviruses (ERVs) occupy 8%–13% of mammalian genomes, while only 2% of those genomes are protein-coding genes [1]. “Endogenization” occurs when exogenous retroviruses integrate into the genomes of germ cells; these ERVs are then inherited by offspring according to Mendelian genetics [2]. The detailed mechanism of by which endogenization occurs is not clearly understood, due to the lack of animal models with which to monitor the endogenization process. As an exceptional case, Tarlinton *et al.* reported that the exogenous koala retrovirus (KoRV) is currently colonizing the koala genome; KoRV is, thus, expected to serve as a good model for understanding endogenization [3]. As with exogenous retroviruses, proviruses of ERVs consist of *gag*, *pol* and *env* genes flanked by long terminal repeats (LTRs) at both ends. Most ERVs are considered to be junk DNA, due to the many deletions and substitutions in their coding sequences; however, recent studies have revealed that several ERVs still retain intact coding sequences and that the encoded proteins are necessary for their host’s physiology [2].

The placenta is a transient organ that appears only during pregnancy and plays essential roles in maintaining the fetus throughout gestation. Recent studies have provided us with evidence that ERVs are involved in placentation in various species [4,5,6,7,8,9,10,11,12]. In this paper, we review recent findings regarding ERVs and placentation, especially in ruminants.

## 2. Diversity of Mammalian Placenta

**Figure 1:**
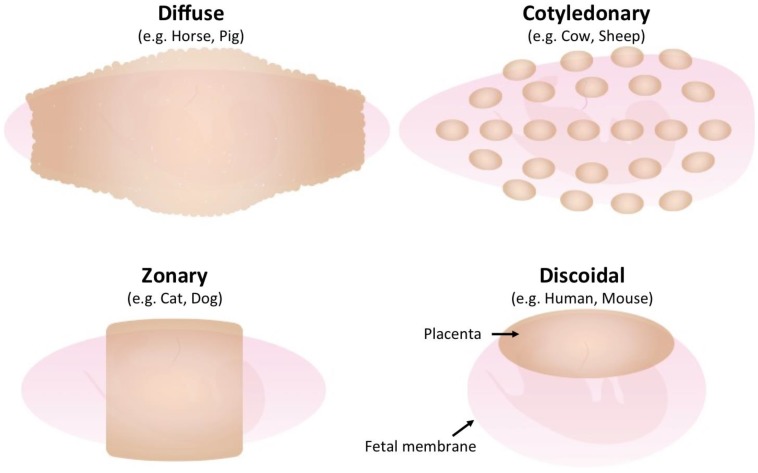
Gross morphology of placentas in different species. The fetus is surrounded by a fetal membrane (light pink). The placenta (light brown) is formed on the fetal membrane and its morphology varies by species.

**Figure 2:**
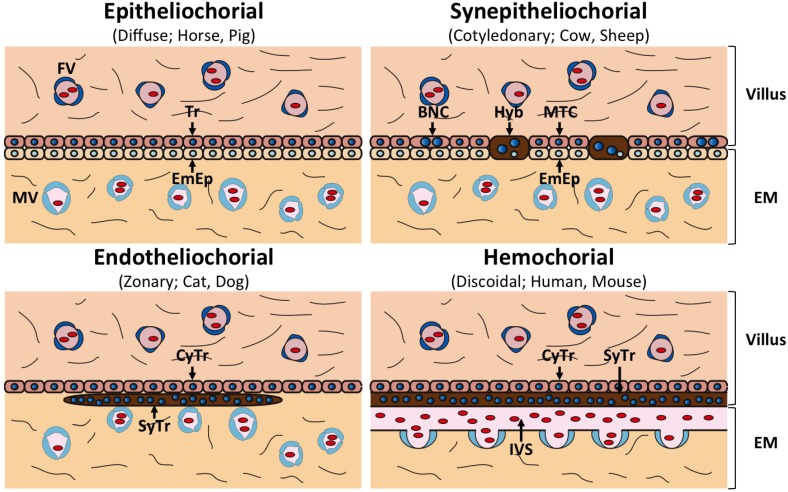
Structure of different types of placentas. The fetomaternal interfaces of the placentas are represented. The endometrial epithelium is retained in epitheliochorial and synepitheliochorial placentas, while it is degraded in endotheliochorial and hemochorial placentas. Abbreviations: FV; Fetal blood vessel, MV; Maternal blood vessel, Tr; Trophoblast, EmEp; Endometrial epithelium, BNC; Binucleate cell, Hyb; Hybrid cell, MTC; Mononucleate trophoblast cell, CyTr; Cytotrophoblast, SyTr; Syncytiotrophoblast, IVS; Intervillous space, EM; Endometrium.

All placentas consist of fetal trophoblast cells and maternal uterine cells; however, placental morphologies differ between species [13]. The gross anatomical morphology of the placenta is divided into four groups: discoidal placenta (e.g., human, mouse), zonary placenta (e.g., cat, dog), cotyledonary placenta (e.g., cow, sheep) and diffuse placenta (e.g., horse, pig) (Figure 1). Each type of placenta has a unique microscopic anatomical feature, namely, hemochorial placenta (discoidal), endotheliochorial placenta (zonary), synepitheliochorial placenta (cotyledonary) and epitheliochorial placenta (diffuse) (Figure 2). The histological classification is defined by the morphology of the fetomaternal interface (Table 1). 

In both hemochorial and endotheliochorial placentas, the trophoblasts deeply invade maternal tissues to allow the efficient exchange of nutrients, gases and hormones. They also form specialized cells called syncytiotrophoblasts, which may prevent rejection of the fetus, the result of maternal immune responses. Syncytiotrophoblasts are formed by continuous cell-to-cell fusion of cytotrophoblasts. Maternal endometrial epithelial cells are completely diminished in these placentas (Figure 2).

The most characteristic feature of synepitheliochorial placentas is that they develop only at projections on the uterine wall, termed caruncles. These placentas are characterized by the presence of about 100 placentomes, which are derived from trophectodermal villi and caruncles, respectively (Figure 1). In synepitheliochorial placentas, the trophoblast cells do not invade the maternal uterine tissue as deeply as occurs in hemochorial and endotheliochorial placentas (Figure 2). At the periimplantation period, the trophoblast cells produce several hormones and proteases that degrade the epithelia of uterine caruncles [14,15,16]. The trophectoderm subsequently adheres to the caruncles and develops the cotyledonary placentomes. The uterine epithelium might be restored after implantation. Some of the trophectodermal cells, termed trophoblast giant cells or binucleate cells (BNCs), fuse with the endometrial cells to form fetomaternal hybrid cells throughout the different stages of pregnancy, including implantation (Figure 2) [17,18]. This fusion is believed to enhance implantation and transport of various substances [17]. This fetomaternal cell-to-cell fusion is the origin of the term “syn” epitheliochorial placenta. 

In contrast, no fused cells appear in epitheliochorial placenta and the trophoblasts do not invade maternal tissues (Figure 2). The fetal and maternal tissues are completely separable in this placenta. 

## 3. ERVs Involved in Placentation

### 3.1. Retroviral Envelope Glycoproteins

The main functions of retroviral envelope glycoproteins (Env) are binding to the entry receptor on the host membrane and the induction of viral-cellular membrane fusion. Retroviral Envs have two subunits: surface (SU) and transmembrane (TM) subunits. The translated Env polyprotein is cleaved into each subunit by cellular proteases, after which SU and TM assemble to establish mature Env trimers of SU-TM heterodimers [19,20,21,22,23,24]. SU recognizes the entry receptors by its receptor binding domains [25,26,27,28,29,30]. The binding of SU leads to conformational changes in Env that expose the fusion peptide at the N-terminus of TM, which then penetrates into the host cell membrane to initiate membrane fusion [31]. Env expression on the cell surface can result in cell-to-cell fusion, as well as viral-cellular membrane fusion; however, the degree of cell-to-cell fusion varies among different retroviral Envs [4,5,6,11,12,32,33].

**Table 1:** Classification of placenta.

Gross Morphology	Microscopic Structure	Species	Fetomaternal Interface	Type of Fused Cells
Diffuse	Epitheliocholial	Horse, Pig	Trophoblast-Epithelial	None
Cotyledonary	Synepitheliocholial	Cow, Sheep	Trophoblast-Epithelial (partially fused)	Fetomaternal hybrid
Zonary	Endotheliocholial	Cat, Dog	Trophoblast-Endothelial	Syncytiotrophoblast
Discoid	Hemochorial	Human, Mouse	Trophoblast-blood	Syncytiotrophoblast

### 3.2. Discovery of Envs Derived from ERVs in Human Placenta

How syncytiotrophoblast morphogenesis occurs was controversial until syncytin-1, the Env of human endogenous retrovirus (HERV) W at 7q21 (ERVW-1), was reported to be involved in the process [4,34]. Although other ERVs (e.g., ERV-3) have also been identified as candidates that might play a role in forming human syncytiotrophoblasts, definitive evidence for their function has not yet been established [35,36,37,38,39]. Syncytin-1 is preferentially expressed in placenta, especially cytotrophoblasts and syncytiotrophoblasts, and has strong fusogenic activity with human trophoblastic cells [4,34]. Syncytin-1 integrated into the common ancestor of hominoids and Old World monkeys (OWMs) between 20 and 30 million years ago (MYA) [40]. While syncytin-1 is believed to contribute to syncytiotrophoblast formation in hominoid placenta, it is inactivated in OWMs that are close relatives of hominoids [40]. Esnault *et al.* recently suggested that OWMs instead use the EnvV syncytin to form syncytiotrophoblasts [41]. They showed that both the *envV1* and *envV2* genes have intact Env coding sequences in primate genomes; however, only EnvV2 had fusogenicity [41]. Both *envV syncytins* were incorporated into the primate genome more than 40 MYA [41]. Even though the *envV2* syncytin is expressed in both OWM and hominoid placenta, the fusogenicity of EnvV2 syncytin has been maintained only in OWMs and not in hominoids [41]. Thus, hominoids and OWMs utilize different ERV Envs for syncytiotrophoblast morphogenesis.

After the discovery of syncytin-1, syncytin-2 was identified as another ERV Env that is specifically expressed in placenta and has fusogenicity [5]. Syncytin-2 appeared in the primate genome more than 40 MYA, as it is found in most primates except for prosimians [5]. While syncytin-1 is preferentially expressed in most syncytiotrophoblast cells, syncytin-2 expression is limited to a few cytotrophoblasts [42,43]. Conversely, the receptors for syncytin-1 (ASCT-2) and syncytin-2 (MFSD2A) are expressed in cytotrophoblasts and syncytiotrophoblasts, respectively [44,45]. Additionally, the fusion activity of syncytin-2 appears to be considerably lower than that of syncytin-1 [33]. Therefore, syncytin-1 and syncytin-2 might have different roles in placentation.

Retroviral Envs also have another important function, namely, immunosuppression. Although the molecular mechanism is not fully elucidated, the leading model suggests that retrovirus-related immunosuppression can be attributed to an immunosuppressive domain (ISD) found in TM [46]. ISD function was first confirmed using a synthetic 17 amino acid-long peptide CKS-17 (LQNRRGLDLLFLKEGGL). This peptide sequence is highly conserved in many retroviruses, including human T-lymphotropic viruses, type C-related HERV, Mason-Pfizer monkey virus, and Syncytins [33,46,47]. Both syncytin-1 and syncytin-2 have ISD motifs; however, only syncytin-2 was able to suppress the immune response *in vivo*, while syncytin-1 was reported to have immunosuppressive activity *in vitro* [33,48,49]. This indicates that syncytin-2 might be an essential piece of the strategy that the placenta adopts to maintain maternal immune tolerance during pregnancy. Taken together, humans and related species might use syncytin-1/EnvV2 and syncytin-2 mainly for forming syncytiotrophoblasts and immunosuppression, respectively.

A number of syncytin-like elements have also been identified in additional species, including rodents (syncytin-A and syncytin-B), lagomorphs (syncytin-Ory1) and carnivores (syncytin-Car1) [7,8,9]. These syncytin-like elements are also believed to form syncytiotrophoblasts. More details regarding these syncytin-like elements are reviewed elsewhere [50].

### 3.3. ERV Envs Expressed in Ruminant Placenta

Three types of trophoblast cells exist in ruminant placenta: mononucleate trophoblast cells (MTCs), BNCs and trinucleate cells (TNCs)/syncytial plaques (SyPs) [17]. Although the exact differentiation process of these cells has not been completely elucidated, a leading model has emerged [18,51]. In this model, BNCs appear as the result of MTC endoreduplication, the replication of genomic DNA without cellular mitosis. The genes for many pregnancy-associated molecules, which have important roles in maintaining pregnancy, are specifically expressed in BNCs and are modulated by epigenetic modification [17,51,52]. The BNCs fuse with maternal endometrial cells to efficiently transfer these molecules to the maternal blood stream [17]. Consequently, fetomaternal hybrid cells appear at the interface of the fetal and the maternal placentomes (Figure 2) [17]. These hybrid cells are categorized as TNCs or SyPs, depending on the species (e.g., Bovinae; TNCs, Caprinae; SyPs) [13,53]. TNCs are composed of a BNC and an endometrial cell, while SyPs are composed of multiple BNCs and an endometrial cell [17,54]. The population of hybrid cells is small and they disappear by apoptosis immediately after exocytosis of the pregnancy-associated molecules [17]. 

For the past decade, it has been suggested that differentiation of ruminant trophoblast cells is driven by ERVs [9,10,11,12,55]. Endogenous Jaagsiekte sheep retroviruses (enJSRVs) are derived from exogenous JSRV and enzootic nasal tumor virus (ENTV), both of which cause tumors in the ovine respiratory tract [56,57,58]. The ovine genome harbors at least 27 enJSRVs and five of them are still intact [57,58]. Some of the enJSRVs are believed to function as restriction factors because they impede the replication of JSRV and ENTV [59,60,61]. In addition, *env* mRNA of enJSRVs named enJS5F16 and enJS56A1 was detected in the ovine placentome, consisting of BNCs, SyPs, and maternal endometrial tissue [9,62]. Dunlap *et al.* demonstrated that inhibition of enJSRV Env expression by morpholino antisense oligos led to death of the conceptus during the periimplantation period due to a failure of BNC development [9]. Even though enJSRV Env is essential for the appearance of BNCs, it remains to be determined whether it is required for the fetomaternal cell-to-cell fusion found in ovine placenta.

**Figure 3:**
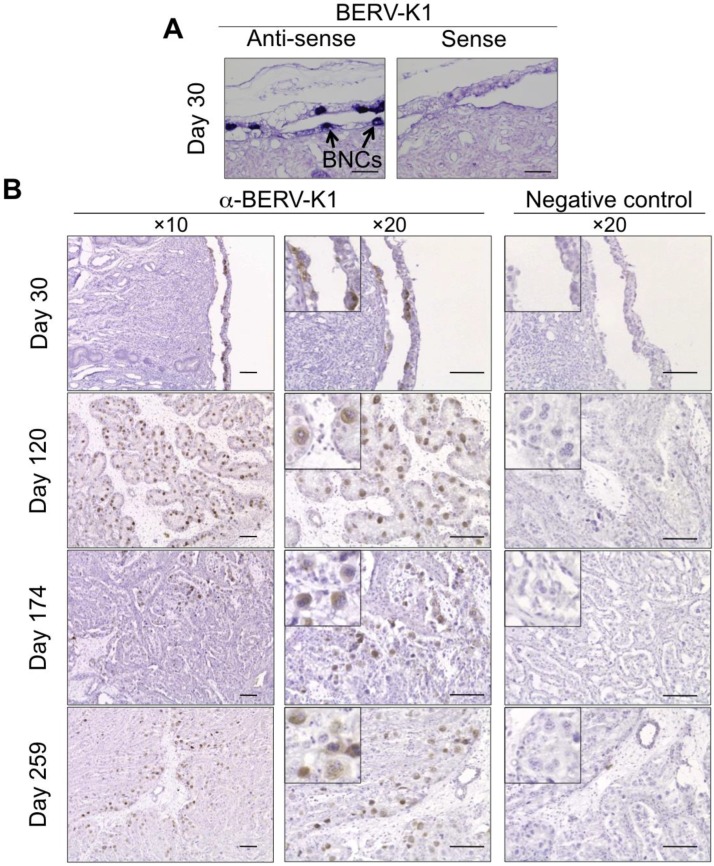
Expression of BERV-K1 envelope in bovine placental tissue. (**A**) *In situ* hybridization of BERV-K1 *env* mRNA on day 30 of gestation in the bovine fetal membrane and maternal endometrium. Scale bars represent 100 μm. DIG-labeled antisense probe for BERV-K1 *env* was used for the hybridization. BNCs express BERV-K1 *env* are shown as blue foci and as indicated by arrows; (**B**) Immunohistochemistry of bovine uterus and fetal membranes on days 30, 120, 174 and 259 gestation. Scale bars represent 100 μm. BNCs are enlarged in small panels. BERV-K1 Env was detected by anti-BERV-K1 Env and appear as brown foci.

Recently, a large number of bovine endogenous retroviruses (BERVs) were identified by various sequencing methods [10,63,64,65,66]. We searched the bovine genome database to identify potential ERV Envs, which could play roles in the fetomaternal cell-to-cell fusion. We identified two candidates, named BERV-K1 and BERV-K2 [10]. Both BERV-K1 and BERV-K2 are categorized as betaretroviruses, which also include JSRV and mouse mammary tumor virus [10,11]. While the BERV-K2 has all of the coding sequences including *gag*, *pro-pol* and *env*, BERV-K1 only retains the *env* coding sequence [10]. We examined the expression of both BERV-K1 and BERV-K2 *env* in bovine organs and found that BERV-K1 *env* but not BERV-K2 *env* was preferentially expressed in bovine placenta [10,11]. This result was further supported by *in situ* hybridization and immunohistochemistry, further demonstrating that BERV-K1 Env alone was detected specifically in BNCs (Figure 3) [11]. We also conducted *in vitro* fusion assays using BERV-K1 Env and primary bovine endometrial cells and found that BERV-K1 Env had high fusogenic activity [11]. This result suggests that BERV-K1 Env induces fetomaternal cell-to-cell fusion at the fetomaternal interface. We also conducted fusion assays using BERV-K2 Env; however, BERV-K2 Env was not able to induce cell-to-cell fusion with endometrial cells [11]. This phenomenon was attributed to a failure of Env glycoprotein maturation [67]. 

BERV-K1 is located in intron 18 of the bovine FAT tumor suppressor homolog 2 (*bFAT2*) gene, which is highly conserved evolutionarily in eukaryotes [11]. We examined other mammalian species for the presence of BERV-K1 by genomic PCR and found BERV-K1 in *Bovinae* (*Bos taurus*, *Bos javanicus*, *Bubalus bubalis*, and *Tragelaphus spekii*) but not other species, including *Caprinae* (*Ovis aries*, *Capra hircus*), human, mouse, dog and cat [11]. From this, we estimate that BERV-K1 infected the common ancestor of *Bovinae* 20 MYA after the separation of *Bovinae* and *Caprinae* [11]. Moreover, BERV-K1 shows evidence of purifying selection (dN/dS < 1) [11]. Thus, we suggested that BERV-K1 Env has a role in forming TNCs in *Bovinae* placenta, although further studies are required to determine its precise function *in vivo*. The BERV-K1 Env was named Fematrin-1 (Fetomaternal trinucleate cell inducer 1) because it is genetically different from syncytins found in the ERVs of other species [11]. We also found that *bFAT2* expression levels were higher in placenta than in other tissues [11]. Additionally, not only *bFAT2* but also ovine *FAT2* was preferentially expressed in placenta [11]. This suggests that the placenta-specific expression of Fematrin-1 is due to its integration into a locus (*bFAT2*), which has high transcriptional activity in ruminant placenta. 

Just prior to our publication describing Fematrin-1, Cornelis *et al.* identified Syncytin-Rum1, also derived from an ERV Env, as a potential factor involved in fetomaternal cell fusion [12]. Syncytin-Rum1 integration occurred over 30 MYA in the common ancestor of ruminants [12]. Syncytin-Rum1 has been subjected to purifying selection and is conserved among most ruminants, with the exception of *Tragulidae* (mouse-deer), whose ruminant ancestor diverged from the others about 50 MYA [12]. Syncytin-Rum1 is expressed in both bovine and ovine placenta and like *Fematrin-1*, was specifically detected in BNCs [12]. Syncytin-Rum1 has the unique characteristic that it is able to exert fusogenicity only under acidic (pH 5.0) but not neutral (pH 7.0) conditions [12]. Hence, the authors suggest that the boundary region of the fetomaternal placenta might be of acidic pH [12]. Further studies regarding whether acidic conditions exist in the ruminant placenta are required to support this hypothesis. Syncytin-Rum1 transcript levels are higher in ovine placenta than bovine placenta throughout gestation [12]. This difference in expression levels between sheep and cattle may be one of the causative agents for the morphological difference in fetomaternal hybrid cells between the two species [12]. 

bERVE-A and BERV-P *env*s have also been identified as transcripts in bovine placenta and trophectoderm [55,63]. The amino acid sequence of bERVE-A is similar to syncytin-1 and is expressed preferentially in BNCs [17]. However, bERVE-A might be the result of BNC appearance rather than an inducer of cell-to-cell fusion because it lacks an intact coding sequence [51]. BERV-P *env* was identified in bovine conceptuses, especially trophectoderm, in the periimplantation period [63]. The amino acid sequence of BERV-P Env is similar to syncytin-Car1 and has both a fusion peptide and an ISD in the TM subunit [63]. However, the functional significance of these elements has not been determined. Table 2 summarizes the placenta-related endogenous retroviral *env* genes that have been identified in ruminants thus far.

**Table 2:** Endogenous retrovirus envelope genes supposed to play roles in placentation in ruminant.

Name of ERV	Species	Expressed Tissues	Expressed Period	Appearance in Host Genome
Syncytin-Rum1	Most ruminants (except for Tragulidae)	Placenta (BNCs)	Through gestation	Over 30 MYA
BERV-K1 (Fematrin-1)	*Subfamily Bovinae*	Placenta (BNCs)	Through gestation	25.4 to 18.3 MYA
bERVE-A	*Bos taurus*	Placenta (BNCs)	Through gestation	Not examined
BERV-P	*Genus Bos*	Trophectoderm	Periimplantation	16.9 to 7 MYA
enJSRV	*Subfamily Caprinae*	Placenta (BNCs), Endometrium	Through gestation	Within 7 MYA

## 4. Perspective on the Relationship between Fematrin-1 and Syncytin-Rum1

As described above, various mammals utilize at least two ERV Envs to induce cell-to-cell fusion in their placenta. However, why mammals utilize multiple ERVs in placental development remains an enigma. Mangeny et al reported that each syncytin has a different primary role, namely either cell-to-cell fusion or immunosuppression, in both human and mouse placentation [33]. In other words, syncytin-1 and syncytin-A have higher fusogenic activity than syncytin-2 and syncytin-B, respectively, while syncytin-2 and syncytin-B have higher immunosuppressive activity [33]. Nakamura and Imakawa previously proposed the “baton pass” hypothesis, in which the roles of either the original host genes or older ERVs have been transferred to newer ERVs [68]. For example, in the case of human syncytins, syncytin-2 probably played roles in both cell-to-cell fusion and immunosuppression at the beginning of endogenization. However, its fusogenic activity would have been gradually attenuated after syncytin-1 integration and ultimately, the main roles of syncytin-1 and syncytin-2 became distinct.

Likewise, a similar situation might be happening in ruminant placenta, at least in the case of the *Bovinae* placenta. Syncytin-Rum1 has both fusogenic and ISD, although the immunosuppressive activity has not been proved [12]. Moreover, syncytin-Rum1 was incorporated into the ruminant genome immediately after the appearance of the ruminant ancestor [12]. In contrast, Fematrin-1 does not contain an ISD, possesses only fusogenic activity and is specific for *Bovinae* [10,11]. We compared the fusogenicity of syncytin-Rum1 and Fematrin-1 in neutral acidic conditions and showed that Fematrin-1 possesses higher activity than syncytin-Rum1 [11]. Thus, we propose that the relationship between syncytin-Rum1 and Fematrin-1 is quite similar to that of human syncytin-1 and syncytin-2: that the role of syncytin-Rum1 (syncytin-2) in cell-to-cell fusion has been being replaced by Fematrin-1 (syncytin-1) in *Bovinae*. Even though no ERVs other than syncytin-Rum1 are known to be involved in cell-to-cell fusion in other ruminants, some as-of-yet unidentified ERV Env(s) or Fematrin-1 could potentially replace, or be in the process of replacing, the cell-to-cell fusion function of syncytin-Rum1. This hypothesis is supported by the observation that syncytin-Rum1 from Giraffidae, Cervidae and some of Bovidae (especially Antilopini) did not show fusogenicity [12]. This suggests that syncytin-Rum1 contributed, in an evolutionary sense, to the appearance of synepitheliochorial placenta and that subsequently, newly acquired ERVs, such as Fematrin-1, further engendered morphological variations in fetomaternal hybrid cells (TNCs or SyPs) in *Bovinae*. Unfortunately, it is difficult to reproduce such ERV-driven ruminant placental evolution, due to the lack of animal models. However, we are able to get an insight into such placental evolution by comparative analysis of ruminant placentas. *Tragulidae* species, like the mouse-deer, might be good animals to study ERV-driven ruminant placental evolution, particularly because their placentas are considered to be a primitive form of the ruminant placenta. Moreover, they are the unique as ruminants, since they lack syncytin-Rum1 [11,12,69].

It is really interesting that most mammals utilize ERV Envs to form fused cells in placentas even though the morphology of their placentas differ. Mammals also seem to have evolved the “baton pass” to employ different ERVs in placentation. This could be considered a kind of convergent evolution among mammals. Comparative studies and mechanistic investigations into ERV-host co-evolution using animal models will bring us new insights into various fields, such as virology and evolutional, reproductive and cell biology.
